# Cardiovascular disease in North African women: insights from the Middle East African Women CardioVascular Disease (MEA-WCVD) registry

**DOI:** 10.3389/fcvm.2025.1577793

**Published:** 2025-09-08

**Authors:** Salma Charfeddine, Leila Abid, Sarra Chenik, Iheb Ben Krayen, Oussama Haddar, Aymen Ghrab, Selim Boudiche, Haithem Touati, Oumaima Ayedi, Mohamed Amine Ammar, Manel Ben Halima, Houssem Ben Ayed, Asma Brahim, Faten ElAyech, Emna Allouche, Houssem Thabet, Houaida Mahfoudhi, Taha Yessine Jabloun, Yasmine Ayadi, Alaeddine Ayadi, Ghassen Romdhani, Hassen Gargouri, Oumayma Zidi, Mohamed Ali Guedri, Rami Tlili, Bechir Trabelsi, Selim Hammami, Rim Othmen, Saoussen Antit, Syrine Saidane, Sirine Dardour, Skander Iddir, Elmahdi Kharrat, Anis Cheikhrouhou, Mohamed Derwich, Amal Mrabet, Taha Lassoued, Emna Rekik, Sahar Gmiha, Niez Laribi, Hakim Lamine, Zied Triki, Samir Ayari, Fatma Boujelbene, Essia Boughzela, Hajer Rekik, Ines Ben Ameur, Syrine Abid, Khalil Oueghlani, Abddayem Haggui, Afef Ben Halima, Wejdene Ouechtati, Emna Bennour, Rania Hammami, Mariem Jabeur, Ihsen Zairi, Mariem Drissa, Faouzi Addad, Sami Milouchi, Mohamed Sami Mourali, Hedi Ben Slima, Leila Bezdah, Elyes Neffati, Youssef Ben Ameur, Sondos Kraiem, Salem Kachboura, Ikram Kammoun, Lilia Zakhama, Hassen lbn Hadj Amor, Khaldoun Ben Hamda, Yosra Messoudi, Nejah Ben Hlima, Rana Dahmani, Habib Gamra, Zied Ibn Elhadj, Hichem Denguir, Chayma Ghorbel, Nizar Mechri, Samia Ernez Hajri, Alexandre Mebazaa, Fedi Ben Dhaou, Maroua Trigui, Wafa Fehri, Salem Abdessalem

**Affiliations:** ^1^ Cardiology Department, Hedi Chaker University Hospital, Sfax, Tunisia; ^2^ Cardiology Department, Military University Hospital, Tunis, Tunisia; ^3^ Cardiology Department, Habib Bourguiba University Hospital, Medenine, Tunisia; ^4^ Cardiology Department, La Rabta University Hospital, Tunis, Tunisia; ^5^ Cardiology Department, Menzel Bourguiba University Hospital, Bizerte, Tunisia; ^6^ Cardiology Department, Charles Nicolle University Hospital, Tunis, Tunisia; ^7^ Cardiology Department, Sahloul University Hospital, Sousse, Tunisia; ^8^ Cardiology Department, Mongi Slim La Marsa University Hospital, Tunis, Tunisia; ^9^ Cardiology Department, Habib Thameur University Hospital, Tunis, Tunisia; ^10^ Cardiology Department, Abderrahman Mami University Hospital, Ariana, Tunisia; ^11^ Cardiology Department, FSI La Marsa University Hospital, Tunis, Tunisia; ^12^ Private Cardiologist, Sfax, Tunisia; ^13^ Private Cardiologist, Tunis, Tunisia; ^14^ Cardiology Department, Djerba Hospital, Medenine, Tunisia; ^15^ Cardiology Department, Taher Sfar University Hospital, Mahdia, Tunisia; ^16^ Cardiology Department B, Fattouma Bourguiba University Hospital, Monastir, Tunisia; ^17^ Cardiology Department, Ibn El Jazzar University Hospital, Kairouan, Tunisia; ^18^ Cardiology Department, Military Hospital, Bizerte, Tunisia; ^19^ Cardiology Department A, Fattouma Bourguiba University Hospital, Monastir, Tunisia; ^20^ Cardiology Department, Taher Maamouri University Hospital, Nabeul, Tunisia; ^21^ Cardiology Department, Gabes Hospital, Gabes, Tunisia; ^22^ Cardiology Department, Kasserine Hospital, Kasserine, Tunisia; ^23^ Cardiology Department, Tatouine Hospital, Tatouine, Tunisia; ^24^ Cardiology Department, Farhat Hached University Hospital, Sousse, Tunisia; ^25^ Department of Anesthesia and Critical Care, Lariboisière Hospital, Paris, France; ^26^ Preventive Department, Habib Bourguiba Hospital, Sfax Tunisia; ^27^ Cardiology Department, Pasteur Clinic, Tunis, Tunisia

**Keywords:** cardiovascular disease, cardiovascular risk factors, women, heart failure, atrial fibrillation, coronary heart disease, valvular heart disease

## Abstract

Cardiovascular disease (CVD) is a major health burden worldwide, yet gender-specific data from the Middle East and North Africa (MENA) region remain scarce. The Middle East African Registry of Women with Cardiovascular Disease enrolled adult patients with coronary heart disease (CHD), heart failure (HF), atrial fibrillation (AF), or valvular heart disease (VHD) across Tunisia between May and July 2023. Of 15,366 patients, 37.6% were women. Compared with men, women were older, had lower socioeconomic status, and presented with more obesity, hypertension, diabetes, dyslipidemia, and sedentary lifestyle but smoked less. CHD was less frequent in women, while AF and VHD were more prevalent. Women underwent fewer coronary angiographies and percutaneous interventions, experienced longer delays, and received fewer guideline-based therapies, including dual antiplatelet agents and high-intensity statins. Among HF patients, women more often had preserved ejection fraction and higher hospitalization rates. These results highlight persistent gender inequities in CVD care in Tunisia.

## Introduction

Numerous studies have uncovered substantial disparities in cardiovascular health between men and women, as well as among different subsets of women (1–4). Despite this, a lack of data exists regarding the distinct features of cardiovascular disease in women within the Middle East North Africa (MENA) region (5, 6). This research gap has led to the underdiagnosis, undertreatment, and under-research of prevalent cardiovascular diseases (CVD), such as coronary heart disease (CHD), including ST-elevation myocardial infarction (STEMI), non-ST-elevation myocardial infarction (NSTEMI), and chronic coronary syndrome (CCS), heart failure (HF) with different phenotypes [preserved ejection fraction (HFpEF), mildly reduced (HFmrEF) or reduced ejection fraction (HFrEF)], valvular heart disease (VHD), and atrial fibrillation (AF), specifically in women.

**Figure 1:**
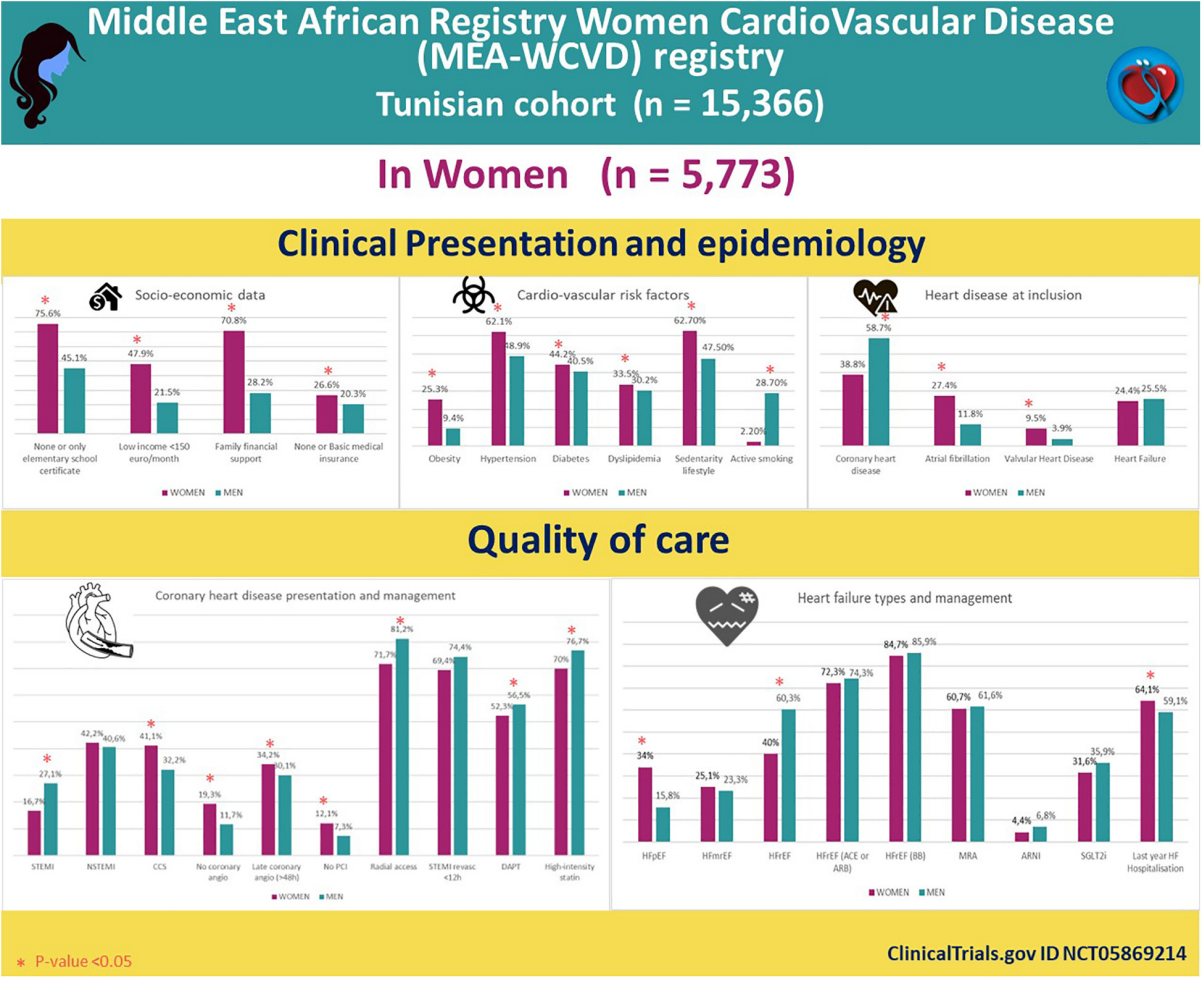
Epidemiology, clinical presentation, and management of the Tunisian cohort in the MEA-WCVD registry.

## Methods

The Middle East African Registry Women CardioVascular Disease (MEA-WCVD) registry is a prospective observational multicentric international study. This publication will focus on the available Tunisian cohort across all governorates of Tunisia, including both public and private health sectors.

The enrolment for the MEA-WCVD registry ClinicalTrials.gov ID NCT05869214 was conducted in Tunisia from 10 May 2023 to 25 July 2023 and involved a one-shot visit.

The study protocol was in accordance with the Helsinki Convention, and the ethical considerations and registry approval were obtained from the south Tunisian Persons' Protection Committee (PPC SUD N°0496/2023) (7).

Eligible patients are all incoming adult patients (≥18 years) with a confirmed diagnosis of the following CVDs: HF, CHD, AF, or VHD, who were admitted to participating centers during the study period.

All patients provided informed consent, and data were collected and stored in a certified health database, managed by our Contract Research Organization (CRO) (Eshmoun, Tunisia). All data were entered into an electronic data capture system with built-in validation rules to minimize entry errors.

Data included patients' demographics, medical history, diagnoses, pharmacological and device therapies focusing on accessibility to health facilities, insurance, time delay to optimal medical therapy, and adherence to guideline-oriented management.

Data were assessed using a standardized questionnaire administered at the time of patient enrolment.

We excluded patients with incomplete medical records or those who declined participation.

The main goal of this paper is to report gender-based disparities in CVD management in Tunisia.

## Results

### Baseline characteristics

Among the 15,366 included patients [CHD *n* = 7,870 (51.2%), HF *n* = 3,857 (25.1%), AF *n* = 2,715 (17.7%), and VHD *n* = 924 (6%)], 37.6% (*n* = 5,773) were female. CHD was significantly lower [2,238 (38.8%) vs. 5,632 (58.7%), *p* ***<*** **10^−3^**], and AF and VHD were significantly higher [AF, 1,580 (27.4%) vs. 1,135 (11.8%), *p* ***<*** **10^−3^**; VHD, 546 (9.5%) vs. 378 (3.9%), *p* ***<*** **10^−3^**] in women and did not differ in HF [1,409 (24.4%) vs. 2,448 (25.5%), *p* = 0.124].

In the overall population, compared with men, women were older {67 years old [IQR = (59–76) vs. 64 years old [IQR = (56–71)], *p* = ***<*10^−3^**}, had lower educational level [none or only elementary school certificate 4,341 (75.6%) vs. 4,313 (45.1%), *p* ***<*** **10^−3^**], lower income (<150 euro per month) [2,752 (47.9%) vs. 2,052 (21.5%), *p* ***<*** **10^−3^**], relied more on financial family support [4,087 (70.8%) vs. 1,746 (28.2%), *p* ***<*** **10^−3^**], and had more basic medical insurance [1,529 (26.6%) vs. 1,940 (20.3%), *p* ***<*** **10^−3^**] (Figure 1).

### Clinical presentation

Regarding CV risk factors (CVRF), women were more obese [1,458 (25.3%) vs. 905 (9.4%), *p* ***<*** **10^−3^**] with higher prevalences of hypertension [3,587 (62.1%) vs. 4,694 (48.9%), *p* ***<*** **10^−3^**], diabetes [2,549 (44.2%) vs. 3,883 (40.5%), *p* ***<*** **10^−3^**], dyslipidemia [1,934 (33.5%) vs. 2,897 (30.2%), *p* ***<*** **10^−3^**], and sedentary lifestyle [<1 h physical activity per week: 3,599 (62.7%) vs. 4,531 (47.5%), *p* ***<*** **10^−3^**] and were less smokers [125 (2.2%) vs. 2,750 (28.7%), *p* ***<*** **10^−3^**]. These differences in CVRF were almost the same in CHD and HF (Figure 1).

### Management and outcomes

Regarding CHD, compared with men, women presented less likely with STEMI [373 (16.7%) vs. 1,520 (27.1%), *p* ***<*** **10^−3^**] and more likely with either NSTEMI [940 (42.2%) vs. 2,276 (40.6%), *p* = 0.198] or CCS [916 (41.1%) vs. 1,812 (32.2%), *p* ***=*** **10^−3^**]. In the acute coronary syndrome (ACS) setting, women were more likely either with no [432 (19.3%) vs. 659 (11.7%), *p* ***<*** **10^−3^**] or delayed time to coronary angiography [**>**48 h: 765 (34.2%) vs. 1,694 (30.1%), *p* ***<*** **10^−3^**] (Figure 1).

Women were less likely to undergo percutaneous coronary intervention (PCI) [no PCI: 45 (12.1%) vs. 111 (7.3%), *p* = ***0.003***] and less radial access [1,598 (71.7%) vs. 4,544 (81.2%), *p* ***<*** **10^−3^**].

Although revascularization delay was almost the same in the STEMI setting [delay <12 h: 258 (69.4%) vs. 1,128 (74.4%), *p* = 0.05], women received less dual antiplatelet therapy (DAPT), less P2Y12 inhibitors [clopidogrel: 1,166 (52.3%) vs. 3,162 (56.5%), *p* ***=*** **10^−3^**] and less high-intensity statins [1,559 (70%) vs. 4,294 (76.7%), *p* ***<*** **10^−3^**].

Regarding HF, compared with men, women presented more likely with preserved ejection fraction [HFpEF: 479 (34%) vs. 388 (15.8%), *p* ***<*** **10^−3^**; HFmrEF: 354 (25.1%) vs. 570 (23.3%), *p* = 0.197; and HFrEF: 563 (40%) vs. 1,477 (60.3%), *p* ***<*** **10^−3^**)], less ischemic etiology [537 (38.1%) vs. 1,497 (60.9%), *p* ***<*** **10^−3^**], and more valvular, hypertensive, and arrythmia causes [161 (11.4%) vs. 188 (7.7%), 268 (19%) vs. 154 (6.3%), and 153 (10.9%) vs. 135 (5.5%), *p* ***<*** **10^−3^** respectively].

In the HFrEF subgroup, guideline-oriented medical therapy compared equally in angiotensin-converting enzyme inhibitors (ACE)/angiotensin receptor blockers (ARB) [407 (72.3%) vs. 1,097 (74.3%), *p* = 0.364], beta-blockers [477 (84.7%) vs. 1,269 (85.9%), *p* = 0.493], and mineralocorticoid receptor antagonists (MRA) [342 (60.7%) vs. 910 (61.6%), *p* = 0.720]. However, access to more costly drugs including sacubitril–valsartan and SGLT2 inhibitors was lower in women [25 (4.4%) vs. 101 (6.8%), ***p*** **=** **0.044**, and 178 (31.6%) vs. 530 (35.9%), *p* = 0.07, respectively].

Women were more prone to have HF hospitalization, the last year before inclusion [360 (64.1%) vs. 873 (59.1%), *p* ***=*** **0.043**].

## Discussion and conclusions

This large Tunisian registry outlined the fact that compared with men, women with CVD presented unexpectedly with more CVRF aside from tobacco. They had low or unstable financial resources or no basic health insurance. In the CHD setting, access to coronary angiography, PCI, and optimal medical therapy was significantly lower and delayed. In the HF setting, compared with the NATURE-HF registry (8), guideline-oriented therapies improved in both genders. New pillars are still less prescribed and especially in women. This may explain the higher rate of HF hospitalization in women with HFrEF.

These recent findings highlight the need for healthcare stakeholders to develop and execute strategies to combat the burden of CVD among women in the MEA region. In Tunisia, several initiatives aim to reduce disparities in cardiovascular care, including national screening programs for hypertension and diabetes, expanded access to primary care in underserved areas, and policy efforts to improve emergency cardiovascular services. However, barriers remain, particularly in access to specialized care and invasive procedures. A national Tunisian registry focusing on secondary cardiovascular prevention with a follow-up of patients with CHD is currently underway, and we intend to report these outcomes in a subsequent publication.

## Data Availability

The raw data supporting the conclusions of this article will be made available by the authors, without undue reservation.
